# Can Bacteria Evolve Resistance to Quorum Sensing Disruption?

**DOI:** 10.1371/journal.ppat.1000989

**Published:** 2010-07-08

**Authors:** Tom Defoirdt, Nico Boon, Peter Bossier

**Affiliations:** 1 Laboratory of Aquaculture and Artemia Reference Center, Ghent University, Gent, Belgium; 2 Laboratory of Microbial Ecology and Technology (LabMET), Ghent University, Gent, Belgium; The Fox Chase Cancer Center, United States of America

## Introduction

**Figure 1:**
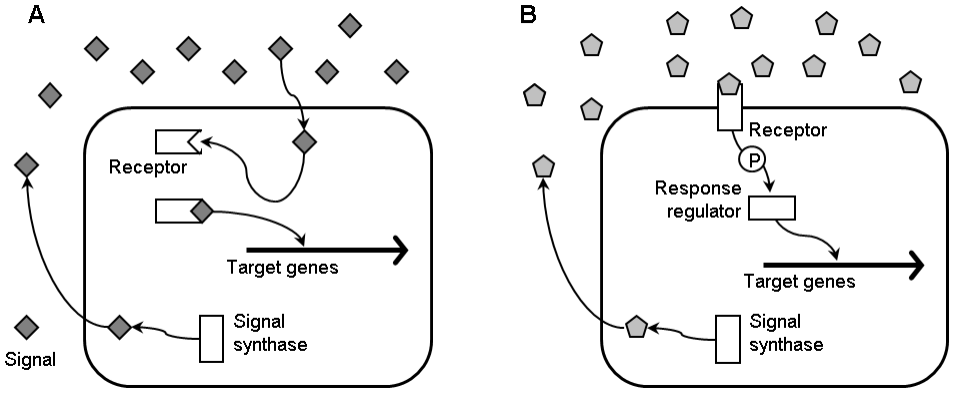
General scheme of a quorum sensing system. The signal synthase enzyme (a homolog of *V. fischeri* LuxI in the case of AHL quorum sensing) produces signal molecules, which reach the extracellular environment either via diffusion or transport. At a critical signal molecule concentration, the signal binds to the receptor, which can be located in the cytoplasm (a homolog of *V. fischeri* LuxR in the case of AHL quorum sensing) (A) or at the cell surface (B). If the receptor is located in the cytoplasm, the signal–receptor complex activates or inactivates transcription of the target genes. If the receptor is located at the cell surface, target gene transcription is modulated through a phosphorylation/dephosphorylation signal transduction cascade with a transcriptional regulator at the end (e.g., a homolog of *V. harveyi* LuxR_Vh_). P denotes phosphotransfer.

Traditional treatment of bacterial infections relies heavily on the use of antibacterial compounds that either kill bacteria (bactericidal) or inhibit their growth (bacteriostatic). Typically, the targets for the main conventional antibiotics are essential cellular processes such as bacterial cell wall biosynthesis, bacterial protein synthesis, and bacterial DNA replication and repair. However, resistance to these drugs arises and spreads very rapidly, even to such an extent that bacteria have been identified that are simultaneously resistant to all available antibiotics [1]. The increasing occurrence of resistant bacteria gradually renders antibiotics ineffective in treating infections and has enormous human and economic consequences worldwide. As a result, the identification of novel drug targets and the development of novel therapeutics constitute an important area of current scientific research. An alternative to killing or inhibiting growth of pathogenic bacteria is the specific attenuation of bacterial virulence, which can be attained by targeting key regulatory systems that mediate the expression of virulence factors. One of the target regulatory systems is quorum sensing (QS), or bacterial cell-to-cell communication. QS is a mechanism of gene regulation in which bacteria coordinate the expression of certain genes in response to the presence or absence of small signal molecules (Figure 1).

## Quorum Sensing: Bacterial Cell-to-Cell Communication

QS was first discovered in the marine bacterium *Vibrio fischeri* and was thought to be restricted to only a limited series of species. Later on, similar systems were found to be present in many other Gram-negative bacteria. These Gram-negative bacteria use acylated homoserine lactones (AHLs) as signal molecules (for a review see [2]). AHLs are typically produced by a homolog of *V. fischeri* LuxI and detected by a homolog of *V. fischeri* LuxR. In addition to the AHL-mediated systems in Gram-negative bacteria, some Gram-positive bacteria also regulate a variety of processes by QS. The QS systems of *Streptococcus pneumoniae*, *Bacillus subtilis*, and *Staphylococcus aureus*, for instance, have been extensively studied (for a review see [3]). A different kind of QS system is found in vibrios. These bacteria use multichannel QS systems in which different types of signal molecules are produced. The signal molecules are detected at the cell surface by membrane-bound, two-component receptor proteins that feed a common phosphorylation/dephosphorylation signal transduction cascade (for a review on QS in vibrios, see [4]). One of the signals produced by vibrios is the so-called autoinducer 2 (AI-2), a furanosyl borate diester [5]. AI-2 activity has been detected in many different species (Gram-negative as well as Gram-positive), although its function as a signal is not generally accepted for all species (for a detailed discussion see [6]). The language of bacteria seems to be even more diversified as new QS systems, using different types of signal molecules, are still being discovered [7].

## Disruption of Bacterial Cell-to-Cell Communication

Phenotypes that are controlled by a QS system include bioluminescence, conjugation, nodulation, swarming, sporulation, biocorrosion, antibiotic production, biofilm formation, and the expression of virulence factors such as lytic enzymes, toxins, siderophores, and adhesion molecules [3], [7], [8]. QS systems are found in a still-growing list of bacteria that are pathogenic to plants, animals, and humans [8], [9]. As the importance of QS in virulence development of pathogenic bacteria became clear, about a decade ago, QS disruption was suggested as a new anti-infective strategy [10].

A first major strategy that has been studied is the application of compounds aiming at interfering with signal molecule detection. The red marine alga *Delisea pulchra* produces halogenated furanones, such as (*5Z*)-4-bromo-5-(bromomethylene)-3-butyl-2(*5H*)-furanone. These compounds disrupt QS-regulated gene expression both in AHL QS systems and in multichannel systems of vibrios by interacting with QS transcriptional regulators [11], [12] and the AI-2 biosynthesis enzyme LuxS [13]. The efficacy of halogenated furanones to protect eukaryotic hosts from animal and human pathogenic bacteria such as *Pseudomonas aeruginosa, Vibrio anguillarum, Vibrio harveyi, Vibrio campbellii*, and *Vibrio parahaemolyticus* has been documented [14]–[16]. Several other macro-algae, micro-algae, and terrestrial plants also produce compounds able to interfere with QS, although in most cases the chemical nature of the signal mimics still has to be elucidated [17], [18]. In addition to these natural compounds, numerous synthetic QS antagonists (mostly AHL and furanone analogs) have been identified and tested (for reviews see [19], [20]).

A second major strategy to disrupt QS is the inactivation of signal molecules. The ability to degrade AHLs seems to be widely distributed in the bacterial kingdom. Enzymes that are able to inactivate AHLs have been discovered in species belonging to the α-proteobacteria, the β-proteobacteria, and the γ-proteobacteria, as well as in some Gram-positive species (for a review see [21]). The actual inactivation of the signal compound can be mediated by two types of enzymes, AHL lactonases and AHL acylases. Lactonases open the lactone ring of AHLs, resulting in the corresponding *N*-acyl-*L*-homoserines, whereas acylases cleave the side chain, releasing homoserine lactone and a fatty acid. Signal-degrading bacteria have been effective against plant, animal, and human pathogens such as *Erwinia carotovora, V. harveyi*, and *Vibrio cholerae* [22]–[24].

Conventional antibiotics inherently favor the evolution of resistance because they pose a strong selective pressure on bacteria. Indeed, resistant mutants have a large fitness advantage when compared to their susceptible counterparts (which are either killed or inhibited in their growth). In this regard, disruption of QS is generally believed to be unlikely to pose harsh selective pressures, thus minimizing the risk of resistance development [14], [15], [20], [25]–[31]. In the following sections, we argue that this point of view might be too optimistic and discuss the possibility that resistance to QS disruption might occur. Although all mechanisms that lead to resistance to conventional antibiotics also apply for QS disruption, the focus will be on variation in the core genes of QS systems (i.e., the genes involved in signal production, detection, and transduction) since this aspect is specific to the possible development of resistance to QS disruption.

## Variability in Quorum Sensing Core Genes

In general, natural selection can only operate on a certain trait if there is (heritable) variation and if this variation is associated with a difference in fitness (i.e., a difference in the amount of offspring that is produced). If these conditions are met, natural selection automatically results. Consequently, there will be a risk for resistance to QS disruption to develop if there is variation in (the expression of) QS genes that can lead to insensitivity towards QS disruption and if this variation results in differences in fitness under QS disrupting conditions. There appears to be variation in the expression of QS core genes among natural bacterial strains. When testing different strains of a certain species for production of QS signal molecules, frequently some of the strains produce signals, whereas others do not. Moreover, variability has been observed in the signal molecule concentration among strains that do produce signals (Table 1). Natural variation in signal molecule levels produced by bacteria might be important when considering QS antagonists that compete with natural signals for receptor binding. Finally, differences among strains of the same species in the specificity of AHL synthases have also been reported. In *E. carotovora* strain SCC3193, for instance, the main AHL is *N*-(3-oxooctanoyl)-*L*-homoserine lactone and only traces of *N*-(3-oxohexanoyl)-*L*-homoserine lactone can be detected, whereas in strain SCC1 an inverse profile is observed [32].

**Table 1:** Examples of inter-strain variability in the production of signal molecules^a^ in different species.

Species	Signal Molecule	ΔActivity (Fold)^b^	n^c^	References
*Aeromonas hydrophila*	BHL and HHL^g^	1.6	4	[33]
*Aeromonas salmonicida*	BHL and HHL^g^	1.4	7^d^	[33]
*Agrobacterium vitis*	long-chain AHLs	15.8	12	[34]
*Burkholderia vietnamiensis*	HHL^g^	2.5	5	[35]
*Erwinia amylovora*	AI-2	2.5	7	[36]
*Fusobacterium nucleatum*	AI-2	9.4	4	[37]
*Photobacterium phosphoreum*	OH-OHL^g^	1.8	3	[38]
*Porphyromonas gingivalis*	AI-2	2.1	6^e^	[37]
*Prevotella intermedia*	AI-2	1.4	7^d^	[37]
*Pseudomonas aeruginosa*	OdDHL^g^	65.5	28^f^	[39]
*Vibrio campbellii*	CAI-1	2.3	7	[40]
	AI-2	2.3	7	
	OH-BHL^g^	2.3	7	
*Vibrio harveyi*	CAI-1	2.0	5	[40]
	AI-2	3.1	5	
	OH-BHL^g^	4.1	5	
*Vibrio salmonicida*	AHL	1.7	8	[33]
*Vibrio vulnificus*	AI-2	5.5	16	[41]

a Zone of induction on agar plate or TLC, or level of induction in liquid assays of a signal molecule reporter strain.

b Ratio between strain-producing maximal and minimal levels, respectively.

c Number of signal-producing strains considered in the calculation.

d Two additional strains were non-producers.

e Three additional strains were non-producers.

f Eight additional strains were non-producers.

g BHL, *N*-butanoyl-*L*-homoserine lactone; HHL, *N-*hexanoyl-*L*-homoserine lactone; OH-OHL, *N*-(3-hydroxyoctanoyl)-*L*-homoserine lactone; OdDHL, *N*-(3-oxododecanoyl)-*L*-homoserine lactone; OH-BHL, *N*-(3-hydroxybutanoyl)-*L*-homoserine lactone.

In addition to differences in the presence and activity of signal molecule synthases, there can also be variation in the presence of signal receptors. In a recent survey of the frequency of AHL-driven QS circuits among genome-sequenced bacteria, differences between different strains of the same species in the number of LuxI and LuxR homologs were reported [42]. In *Burkholderia mallei*, for instance, the number of LuxR homologs varied from two to five. Moreover, a study by Zhu and co-workers indicated that bacteria could simply circumvent the QS blockade by overexpressing signal molecule receptor genes. Indeed, many synthetic AHL analogs were potent QS inhibitors in wild-type *Agrobacterium tumefaciens* [43], whereas in a transformed strain that overexpressed the *luxR* homolog *traR*, inhibition was not detected for any of the analogs [43]. Finally, changes in the specificity of the receptor might also affect the outcome of QS disruption. Indeed, a point mutation of L42→A in the LuxR signal binding site has been shown to render the receptor insensitive to the synthetic antagonist *N*-(propylsulfanylacetyl)-*L*-homoserine lactone, which even served as an agonist for this mutant [44]. Importantly, although the mutation rendered the signal receptor insensitive to the inhibitor, it maintained wild type sensitivity to activation by the natural signal.

Variation in QS signal transduction genes has also been documented. Joelsson and co-workers surveyed the QS systems of different *V. cholerae* strains and observed an unexpectedly high rate of dysfunctional components [45]. Some of the strains showed constitutive expression of QS-regulated genes, and others had frame shift mutations in *hapR*, a partial deletion in *hapR*, or even no *hapR*, resulting in non-functional QS regulation. Interestingly, Defoirdt and co-workers observed differences between closely related vibrios with respect to halogenated furanone-mediated protection of infected brine shrimp larvae [16]. This might reflect differences between the strains in production levels, sequence, or structure of the master regulator LuxR_Vh_, the target of the furanone [12].

Differences between strains in the presence and activity of QS core genes can be caused by horizontal gene transfer. Indeed, the *traRI* operon (encoding the LuxR and LuxI homologs TraR and TraI) of the plant pathogen *Agrobacterium tumefaciens* is located on the Ti plasmid [46]. In an exciting report, Wei and co-workers identified a functional QS system in *Serratia marcescens* that is carried on a transposon [47]. The acquisition of such a mobile QS system might enable bacteria to circumvent specific disruption of their native QS system, provided that the new signal–receptor complex is able to activate target gene expression. Interestingly, Coulthurst and co-workers reported that transfer of the *Serratia marcescens smaIR* operon (encoding homologs of *V. fischeri* LuxI and LuxR) into the QS-deficient strain *Sma* 274 caused a variety of native traits, including pigment production, to become QS regulated [48]. These results suggest that QS core genes can indeed be “plugged into” a strain's existing regulatory systems.

## Effect of Quorum Sensing Disruption on Fitness

In the previous section, we provided an overview of data indicating that variation in QS core genes that could result in insensitivity to QS disruption exists or can originate easily (by point mutation). A second important question to answer is whether this insensitivity could lead to increased fitness under QS-disrupting conditions. It is thus critical to correctly evaluate the effect of QS disruption on the fitness of bacteria in order to accurately predict the risk of resistance development. Many reports have shown that QS does not affect bacterial growth [14], [15], [25], [28], [29], and therefore it is generally believed that QS disruption only has a small or even no effect on fitness. However, all these observations were made under conditions where bacteria were growing in nutrient-rich synthetic growth media (where QS-regulated genes are not essential for growth). Importantly, as pointed out by Martinez and colleagues, a crucial and underappreciated aspect of fitness measurements is that they must be performed under conditions that are as similar as possible to the clinical situation [49]. Hence, the question that arises is whether QS disruption poses selective pressure on the bacteria where it really matters—in vivo during infection. If it does, then a mutant that is insensitive to QS disruption will have a selective advantage over the (sensitive) wild type and resistance will develop.

Imamura and co-workers reported that the numbers of viable *P. aeruginosa* PAO1 in the lungs of infected mice in a respiratory infection model were 3.3-fold lower for the *rhlI* mutant than for the wild type 2 weeks after infection [50]. Similarly, the levels of *P. aeruginosa* PAO1 were 3 log units lower in mouse lungs treated with a QS-disrupting furanone when compared to untreated mice in an injection model of infection [14]. According to the authors, this decrease was due to increased clearance of the bacteria by the mouse immune system. However, it might as well reflect a decreased ability of the pathogen to colonize the host. Indeed, Lesic and coworkers found that *P. aeruginosa* cell counts at the site of infection were not affected by QS inhibitors in a mouse burn wound infection model, whereas cell counts in adjacent muscle and blood were 2–3 log units lower [51]. This indicated that the QS inhibitors blocked systemic dissemination of the pathogen. From the perspective of the bacteria, both increased clearance or decreased colonizing ability would lead to a decrease in fitness, and consequently, a mutant that is insensitive to QS disruption would have a selective advantage over the susceptible wild type (because the capability to colonize the host is undisturbed in the mutant and/or because the host immune system is unable to eliminate the mutant).

It appears that under certain conditions, QS disruption can indeed affect bacterial growth. In a highly interesting report, Diggle and co-workers studied the growth of *P. aeruginosa* wild type and *lasI* and *lasR* mutants in different environmental conditions. In nutrient-rich medium, QS-deficient mutants reached a 1.5-fold higher cell density than the wild type, indicating that under these conditions the costs of signalling were higher than the benefits [52]. In contrast, in a medium in which QS-regulated protease expression is needed for growth, the growth of the mutants was 3- to 4-fold lower than that of the wild type. Hence, under these conditions, QS disruption appeared to strongly decrease the fitness of the pathogen [52]. Under in vivo conditions (i.e., during infection of a host), the fitness advantage of QS might be less pronounced than in the growth medium where QS was essential for growth. In vivo, there will be different nutrient sources present (protein, lipids, phospholipids) and the utilization of some of these nutrients will probably not be controlled by QS. Moreover, the selective pressure for resistance development to QS disruption will be limited to those environmental conditions in which the QS-regulated genes affect bacterial fitness. This is in contrast to conventional (bactericidal and bacteriostatic) antibiotics, which pose strong selective pressure in any environment.

The above mentioned results are in accordance with the work of Sandoz and colleagues, who studied social cheating in *P. aeruginosa* and found that a *lasR* mutant was unable to grow in medium containing caseinate as the sole carbon source [53]. Although this cheater mutant showed higher fitness than the wild type when co-cultured in this medium (the mutant could benefit from the nutrients generated by the proteolytic activity of the wild type), total culture density decreased with increasing amounts of mutant cells. This indicated that the presence of the mutants did incur a significant cost to the population as a whole. The authors also reported that during an in vitro evolution experiment in which wild type and *lasR* mutant were co-cultured for ≈100 generations under conditions that require QS for growth, compensatory mutations emerged that converted cheaters into cooperators. In these novel cooperators, the compensatory mutation resulted in the expression of the QS-regulated phenotype in the *lasR* mutants, thereby bypassing inactivation of the QS system. The evolution of a cheater to a superior cooperator has also been reported in the fruiting body–forming bacterium *Myxococcus xanthus* [54]. The capacity for compensatory mutation could be a mechanism of bacteria to overcome QS disruption and as such might be important for possible resistance development.

There is some evidence that QS might affect the elimination of bacteria by the host immune system. Joelsson and co-workers reported that QS enhances the viability of *V. cholerae* under stress conditions in a HapR-dependent manner [55]. Similarly, McDougald and co-workers found that QS induces stress resistance in *Vibrio angustum* and *Vibrio vulnificus* [56]. Inactivation of the QS master regulator SmcR in *V. vulnificus* resulted in a significantly decreased survival after exposure to hydrogen peroxide, which is a part of the defense of eukaryotic hosts against infections [57]. Hence, QS disruption leading to an increased susceptibility to oxidative immune reactions of the host will reduce the fitness of a pathogen under in vivo conditions.

## Conclusions and Further Perspectives

QS disruption has been shown to be an effective anti-infective strategy in different host–microbe systems and is therefore considered to be a promising alternative to antibiotics. It is generally believed (although yet not proven) that pathogens are unlikely to develop resistance to this strategy because it poses no or little selective pressure. In this paper, we critically evaluated the information that is available on competition/adaptive evolution of QS mutants in order to obtain a more balanced view. A number of studies in which QS was investigated under conditions that are different from those in standard laboratory cultures using nutrient-rich synthetic growth media and that are more representative of the conditions pathogens experience during infection of a host indicate that—in contrast to the general perception—disruption of QS can pose selective pressure on bacteria. Hence, although at this moment it is difficult to accurately estimate the risk of resistance development, we argue that scientists need to pay attention to the possibility that it will evolve. Further research in different host–microbe systems is urgently needed in order to obtain a more detailed understanding of the fitness cost of QS disruption under in vivo conditions during infection of a host. In this respect, in vivo competition experiments with wild types versus QS mutants of pathogenic bacteria in infection models with a susceptible host would give highly relevant information on selective pressure posed by QS inhibition under in vivo conditions. In addition to this, in vivo evolution experiments in which QS regulation of virulence is studied over many generations during infection of a host under QS disruption conditions would give direct information on the risk of resistance development. To this end, the pathogen could be re-isolated from the infected host after each round of infection to be used as inoculum for the next round.

Once we have better knowledge of the risk of resistance development to QS disruption, it might be possible to direct further research on QS inhibition preferentially towards strategies that include a lower risk of resistance development. A first strategy might consist of using QS disrupting techniques with a relatively broad activity. AHL lactonase, for instance, is active towards a wide range of AHLs. It hydrolises both short- and long-chain AHLs with similar efficiency, but shows no or little residue activity to other chemicals, including non-acyl lactones and aromatic carboxylic acid esters [58]. Hence, alterations of the type of AHL will not affect the efficacy of lactonases. Apart from that, algae and higher plants have been found to produce several different compounds that interfere with QS, and thus the application of plant or algal extracts or exudates might also reduce the risk of resistance development. The red marine alga *D. pulchra*, for instance, produces several different, but structurally related, brominated furanones [59]. The production of different QS inhibitory compounds might be an evolutionary adaptation that avoids resistance development by fouling bacteria. Indeed, although there are approximately a million different bacterial species in the marine environment [60], none have developed resistance to the collection of furanones produced by *D. pulchra* since the alga is not colonized by bacteria. Another strategy might be the development of non-competitive or uncompetitive inhibitors rather than competitive inhibitors. Such inhibitors would not suffer from titration effects due to overexpression of QS core genes (e.g., differences between strains in the production of signal molecules). Further, QS disruption could be combined with other treatments to obtain a synergistic effect. QS-disrupting compounds have been shown, for instance, to increase the susceptibility of biofilm bacteria for antibiotic treatments [14]. Finally, the major virulence factors responsible for infection of the host could be targeted directly instead of blocking their expression by QS disruption (for reviews on this strategy, see [61], [62]). However, resistance to this strategy might also evolve if the virulence factor that is inactivated affects pathogen fitness under in vivo conditions.
